# Short-term associations between ambient air pollution and cardiovascular disease mortality: an 11-year time-series study in Nanning, China

**DOI:** 10.3389/fpubh.2026.1878398

**Published:** 2026-07-15

**Authors:** Xia Luo, Linhan Liang, Yang Yu, Zhu Liang, Huiting Nong, Dan Gu, Li Chen, Zhi Li, Lin Ye, Huanhuan Chen

**Affiliations:** 1 Institute of Environmental Health and Endemic Disease Control, Guangxi Zhuang Autonomous Region Center for Disease Control and Prevention (Guangxi Zhuang Autonomous Region Academy of Preventive Medicine), Nanning, China; 2 Department of Microbiology, School of Basic Medicine, Guangxi Medical University, Nanning, Guangxi, China; 3 Division of Chronic Disease Control and Prevention, Nanning Center for Disease Control and Prevention, Nanning, China

**Keywords:** ambient air pollution, cardiovascular disease, daily mortality, time-series study, vulnerable populations

## Abstract

**Background:**

Short-term exposure to ambient air pollution has been associated with cardiovascular disease (CVD) mortality, but localized evidence from subtropical cities in southwestern China remains limited. This 11-year time-series study aimed to evaluate the short-term associations between six criteria air pollutants and daily CVD mortality in Nanning, China.

**Methods:**

We conducted a time-series analysis using daily data on CVD mortality, air pollutant concentrations, and meteorological variables from 2014 to 2024 in Nanning. Generalized additive quasi-Poisson models were used to estimate associations across single-day lags (lag0-lag7) and cumulative lag windows (lag0-1 to lag0-7), adjusting for long-term trends, temperature, relative humidity, and day of the week. Exposure-response relationships were assessed using natural cubic splines. Stratified analyses were conducted by season and age group, and two-pollutant models and sensitivity analyses were performed to evaluate robustness.

**Results:**

A total of 87,913 CVD deaths were recorded during the study period, of which 57.5% occurred in males and 77.9% occurred among individuals aged ≥65 years. In single-day lag models, PM_2.5_, PM_10_, SO_2_, NO_2_, and O_3_-8h showed significant positive associations with CVD mortality at early lag days. At lag0, the RRs were 1.014 (95% CI: 1.008–1.020) for PM_2.5_, 1.009 (95% CI: 1.006–1.013) for PM_10_, and 1.018 (95% CI: 1.011–1.026) for NO_2_, while SO_2_ showed its largest estimate at lag1 [RR = 1.044 (95% CI: 1.018–1.069)]. In cumulative lag models, PM_2.5_, PM_10_, SO_2_, NO_2_, and O_3_-8h showed significant cumulative associations, whereas CO was significant mainly in shorter lag windows. Season-stratified analyses suggested pollutant-specific variation between cold and warm seasons, although formal pollutant-by-season interaction tests were not statistically significant. Age-stratified analyses indicated broader susceptibility among older adults. The main associations were generally robust in sensitivity analyses, distributed lag models, and two-pollutant models, particularly for PM_2.5_, PM_10_, and NO_2_.

**Conclusion:**

Short-term exposure to ambient air pollution was associated with increased CVD mortality, particularly for PM_2.5_, PM_10_, and NO_2_, with consistent effects across single-day and cumulative lag structures. The results highlight the cardiovascular risks of particulate and traffic-related pollution and support pollutant-specific air quality management and targeted protection for older adults during high-pollution periods.

## Introduction

Ambient air pollution is a leading global environmental risk factor for premature mortality. According to the Global Burden of Disease Study, ambient particulate matter pollution alone was responsible for approximately 6.67 million premature deaths in 2019, ranking it among the foremost global risk factors (1). CVD is the dominant health outcome, accounting for about 60% of these air pollution-attributable deaths (2).

Robust epidemiological evidence indicates that short-term exposure to multiple ambient air pollutants significantly increases the risks of morbidity and mortality at the population level. The causal relationship between fine particulate matter (PM_2.5_), its co-emitted inhalable fraction (PM_10_), and cardiopulmonary mortality is most extensively established (3, 4). Moreover, key components within this complex pollutant matrix, including sulfur dioxide (SO_2_), nitrogen dioxide (NO_2_), ozone (O_3_-8h), and carbon monoxide (CO), have been consistently implicated in diverse adverse health effects (5–8). While these gaseous pollutants often share common sources with particulates, their health effects are likely mediated through distinct pathophysiological pathways (9, 10). Therefore, a systematic evaluation of the independent and joint health effects of multiple pollutants within a specific regional pollution context is essential for quantifying the local disease burden and informing targeted control strategies. Time-series analysis is a key methodological tool for quantifying these acute effects. Large-scale multi-city studies have consistently demonstrated positive associations between daily variations in pollutant concentrations and daily CVD mortality and morbidity (11–13). These analyses have also revealed significant heterogeneity in effect estimates. Older adults exhibit heightened susceptibility, largely due to diminished physiological reserve and a higher burden of pre-existing conditions (14, 15). Similarly, health effects are typically more pronounced during cold seasons (16, 17). Elucidating pollutant-specific lag patterns and exposure-response relationships is therefore fundamental for precise risk assessment.

Despite this substantial body of evidence, important knowledge gaps remain. First, high-quality epidemiological studies are predominantly concentrated in northern and eastern urban agglomerations in China. There is a notable paucity of long-term, systematic assessments for specific regions such as Guangxi in southwestern China, which is characterized by a subtropical climate, rapid urbanization, and ongoing energy transition. Second, within the realistic context of multi-pollutant coexistence in such understudied regions, the independent health contributions of key pollutants like PM_2.5_ and NO_2_, as well as the quantitative modification of their effects by season (cold vs. warm) and age (older adults [≥65 years] vs. adults aged [<65 years]), remain poorly characterized. This lack of localized quantitative evidence hinders the development of targeted public health interventions. Therefore, this time-series study covering the period from 2014 to 2024 in Nanning, Guangxi, aimed to quantify the short-term effects of six criteria pollutants on CVD mortality, characterize exposure-response relationships, and examine effect modifications by season and age.

## Materials and methods

### Study location

This study was conducted in Nanning, the capital city of the Guangxi Zhuang Autonomous Region, China (22°13′–23°32′N, 107°45′–108°51′E). Characterized by a subtropical monsoon climate, Nanning is a major transportation hub and industrial center in southern China, with an urban population exceeding 8 million. Its distinct climatic conditions, rapid urbanization, and complex emission profiles make it a representative area for assessing the health effects of air pollution in subtropical cities.

### Data sources

#### Daily mortality data

Daily CVD mortality data for Nanning residents from January 1, 2014, to December 31, 2024, were obtained from the Nanning Center for Disease Control and Prevention (CDC) vital registration system. Cause of death was classified according to the International Classification of Diseases, Tenth Revision (ICD-10). All deaths with underlying causes coded as I00–I99 (diseases of the circulatory system) were included. A total of 87,913 CVD deaths were recorded during the study period. The population at risk included registered residents of Nanning, and each death certificate represented a unique individual death record coded according to standardized national ICD-10 procedures throughout 2014–2024.

#### Ambient air pollution data

Air pollution data were acquired from the Nanning Environmental Monitoring Center. The data included 24-h average concentrations of PM_2.5_, PM_10_, SO_2_, NO_2_, CO, and O_3_-8h. Data were collected from state-controlled air quality monitoring stations evenly distributed across the urban built-up area of Nanning, covering residential, commercial, traffic, and mixed zones to effectively represent population exposure. After quality control, the arithmetic mean of all valid station data was calculated as the city-wide daily average concentration.

#### Meteorological data

Daily meteorological data, including daily mean temperature and daily mean relative humidity for the corresponding period, were provided by the Nanning Meteorological Bureau. These variables were utilized to control for the influence of meteorological conditions in the statistical models.

### Statistical analysis

A time-series analysis was performed using generalized additive models (GAMs) with a quasi-Poisson distribution to assess the short-term association between air pollutant exposure and daily CVD mortality. This method effectively handles overdispersion in count data and flexibly controls for nonlinear confounders (18, 19, 35). The basic model was specified as follows:

$$\begin{array}{l} \log[E(Y\_t)]=\alpha +\beta \times \text{Pollutant}\_\{t,l\}+\mathrm{ns}(\text{time},\mathrm{df}=7/\text{year})+\\ \;\;\;\;\;\;\;\;\;\;\;\;\;\;\;\;\;\;\;\;\;\;\;\;\;\;\;\;\;\;\;\;\;\;\;\;\;\;\mathrm{ns}(\text{temperature},\mathrm{df}=5)+\\ \;\;\;\;\;\;\;\;\;\;\;\;\;\;\;\;\;\;\;\;\;\;\;\;\;\;\;\;\;\;\;\;\;\;\;\;\;\;\mathrm{ns}(\text{relative humidity},\mathrm{df}=4)+\mathrm{DOW}\_t \end{array}$$

where E(Y_t) denotes the expected number of deaths on day t; α is the intercept; β represents the log-relative risk associated with a unit increase in pollutant concentration at lag l; Pollutant_{t,l} is the pollutant concentration on day t at lag l; ns() denotes a natural cubic spline smoothing function; and DOW_t is a factor variable for day of the week.

Associations were expressed as percent excess risk (ER%) and 95% confidence intervals (CIs). For PM_2.5_, PM_10_, SO₂, NO₂, and O_3_-8h, ER% was calculated per 10 μg/m^3^ increase as ER% = [exp (*β* × 10) − 1] × 100%. For CO, ER% was calculated per 0.1 mg/m^3^ increase as ER% = [exp (*β* × 0.1) − 1] × 100%.

To comprehensively characterize the associations, we conducted a series of systematic analyses. First, lag effects were assessed using both single-day lag models from lag0 to lag7 and cumulative lag models from lag0–1 to lag0–7. Second, nonlinear exposure-response relationships were explored by applying a natural cubic spline with 5 degrees of freedom to the pollutant concentration term, using the 5th percentile of each pollutant distribution as the reference. Third, pre-specified stratified analyses were performed to examine potential effect modification by season and age group. Season was defined *a priori* using a calendar-based classification, with November to April classified as the cold season and May to October classified as the warm season. Formal pollutant × season interaction tests were conducted by adding cross-product terms between each pollutant and season to the generalized additive quasi-Poisson models. Age-stratified analyses were performed for individuals aged <65 and ≥65 years. Fourth, to evaluate the robustness of pollutant-specific associations in multi-pollutant settings, two-pollutant models were constructed by incorporating the main pollutant and one co-pollutant simultaneously into the same model. Highly correlated pollutant pairs were not simultaneously included, and collinearity diagnostics, including variance inflation factors and condition indices, were used to assess model stability. Several sensitivity analyses were further conducted to evaluate the robustness of the findings. First, we varied the degrees of freedom for the long-term time trend from 4 to 10 per year and adjusted the smoothing specifications for temperature and relative humidity. Second, we additionally adjusted for public holidays, Chinese New Year, and the COVID-19 pandemic period. Third, false discovery rate correction was applied to the main lag analyses to account for multiple testing. Fourth, distributed lag models were fitted as supplementary analyses to evaluate the lag structure within a unified modeling framework. Finally, to assess potential mortality displacement, the lag window was extended to lag0–14 and lag0–21, and the cumulative estimates were examined to determine whether early positive associations were attenuated or followed by negative compensatory effects at later lags. All analyses were conducted using R software (version 4.5.1), with statistical significance set at a two-sided *p*-value <0.05.

## Results

### Descriptive characteristics of the study population and pollutants

**Figure 1:**
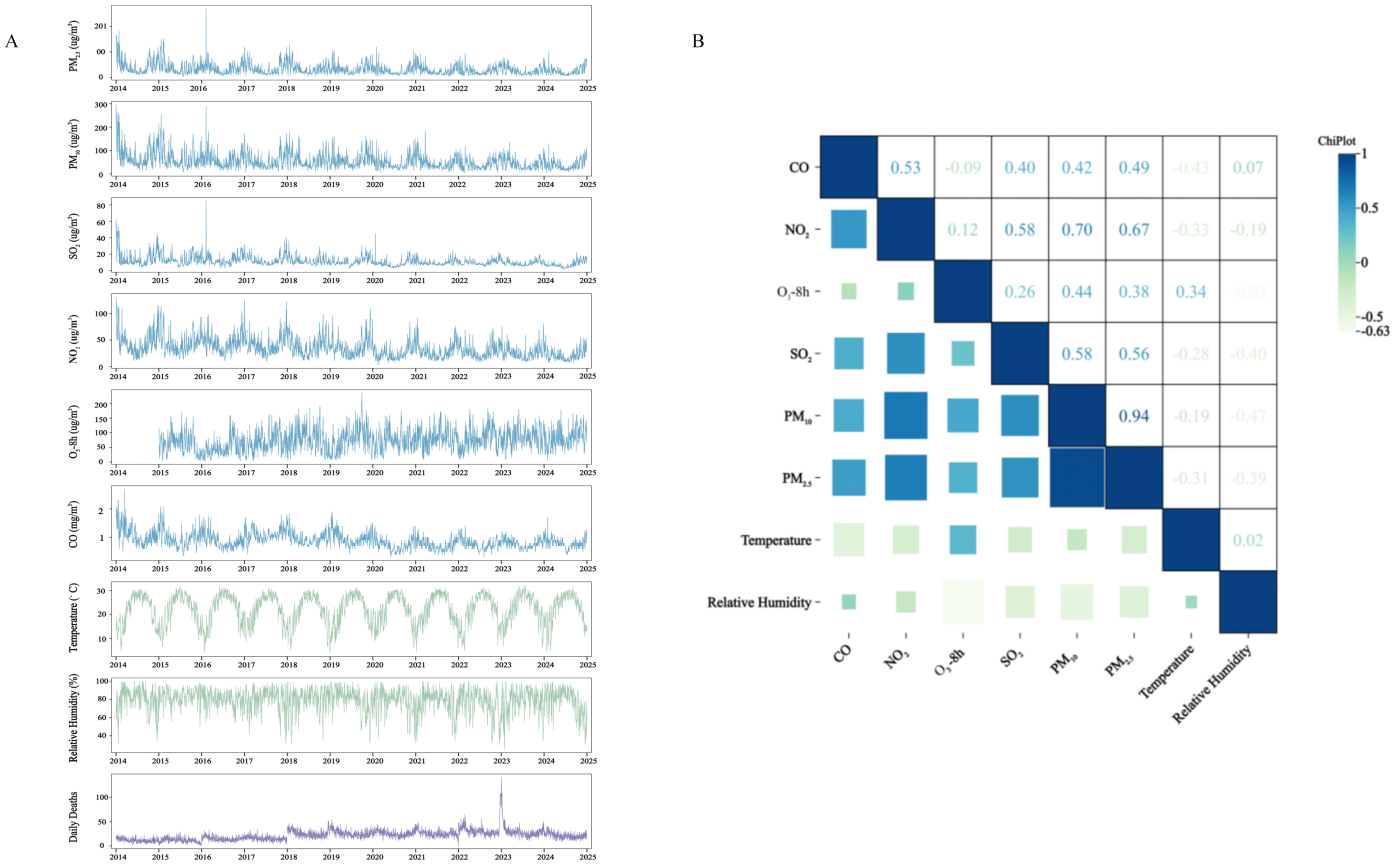
**(A)** Time-series of air pollutant concentrations, meteorological conditions, and daily counts of cardiovascular disease mortality. **(B)** Spearman’s correlation coefficients between air pollutants and meteorological variables.

A total of 87,913 CVD deaths were recorded in Nanning from 2014 to 2024. The deceased were predominantly male (57.5%) and aged ≥65 years (77.9%) (Table 1). As summarized in Table 2, the mean daily concentrations of the major pollutants varied substantially during the study period. The mean daily concentrations were 31.0 μg/m^3^ for PM_2.5_, 51.3 μg/m^3^ for PM_10_, 33.3 μg/m^3^ for NO_2_, 10.9 μg/m^3^ for SO_2_, 74.4 μg/m^3^ for O_3_-8h, and 0.9 mg/m^3^ for CO. The mean daily temperature was 22.2 °C, and the mean relative humidity was 79%. Figure 1A shows clear seasonal patterns for PM_2.5_, PM_10_, and SO₂, with higher concentrations during the cooler months. Daily CVD deaths remained relatively stable between 2014 and 2022 but showed a marked increase in 2023.

**Table 1:** Demographic characteristics of the study participants.

Variables	Statistic *n* (%)
Total	87,913 (100.0)
Sex, *n* (%)	
Male	50,575 (57.5)
Female	37,338 (42.5)
Years, *n* (%)	
<65	19,449 (22.1)
≥65	68,464 (77.9)

**Table 2:** Characteristics of air pollutants and meteorological factors for study participants.

Variables	Mean ± SD	Min	Median	Max
Air pollutants				
NO_2_ (μg/m^3^)	33.3 ± 17.2	3	30	125
SO_2_ (μg/m^3^)	10.9 ± 5.1	1	8	85
O_3_-8h (μg/m^3^)	74.4 ± 36.3	3	72	236
PM_10_ (μg/m^3^)	51.3 ± 30.2	4	44	290
PM_2.5_ (μg/m^3^)	31 ± 19.8	3	26	269
CO (mg/m^3^)	0.9 ± 0.2	0.3	0.8	2.08
Temperature (°C)	22.2 ± 6.3	3.8	23.8	32.3
Relative humidity (%)	79 ± 12.8	25	82	100

### Intercorrelations among air pollutants and meteorological factors

The Spearman correlation matrix (Figure 1B) summarizes the relationships among the six air pollutants and two meteorological variables. PM_2.5_ and PM_10_ were highly correlated (*r* = 0.94), representing the strongest correlation in the matrix. NO_2_ was also strongly correlated with PM_10_ (*r* = 0.70) and PM_2.5_ (*r* = 0.67). SO_2_ showed moderate positive correlations with both NO_2_ (*r* = 0.58) and PM_10_ (*r* = 0.58). CO was moderately correlated with NO_2_ (*r* = 0.53) and PM_2.5_ (*r* = 0.49). In contrast, O_3_-8h showed a distinct pattern, with a weak negative correlation with CO (*r* = −0.09), a weak positive correlation with NO_2_ (*r* = 0.12), and moderate positive correlations with PM_10_ (*r* = 0.44) and PM_2.5_ (*r* = 0.38). Temperature was negatively correlated with most pollutants, particularly CO (*r* = −0.43), NO_2_ (*r* = −0.33), and PM_2.5_ (*r* = −0.31), but positively correlated with O_3_-8h (*r* = 0.34). Relative humidity was strongly negatively correlated with O_3_-8h (*r* = −0.63) and moderately negatively correlated with PM_10_ (*r* = −0.47) and SO_2_ (*r* = −0.40), whereas its correlation with PM_2.5_ was weakly positive (*r* = 0.07).

### Associations between air pollutants and CVD mortality

**Figure 2:**
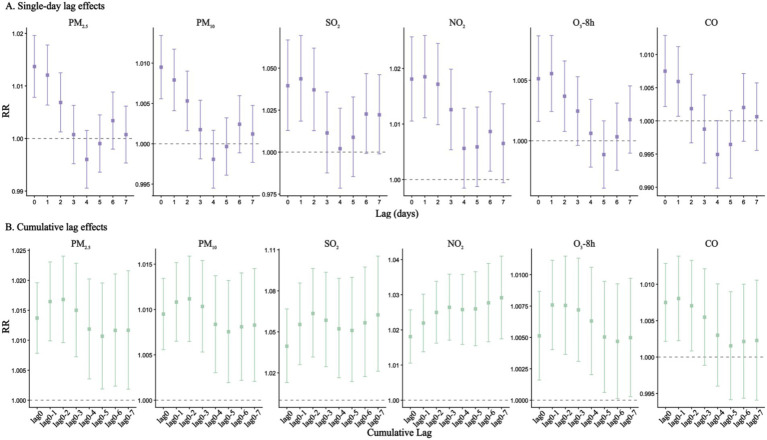
Relative risks and 95% confidence intervals for cardiovascular mortality associated with air pollutants across different lag days. **(A)** Single-day lag effects from lag0 to lag7. **(B)** Cumulative lag effects from lag0–1 to lag0–7. Estimates are expressed per 10 μg/m³ increase in PM_2.5_, PM_10_, SO_2_, NO_2_, and O_3_-8h, and per 0.1 mg/m^3^ increase in CO.

In the single-day lag models (Figure 2A), most pollutants showed positive associations with CVD mortality at early lag days, although the lag patterns differed across pollutants. For PM_2.5_, the association was statistically significant from lag0 to lag2, with the strongest estimate at lag0 [RR = 1.014 (95% CI: 1.008, 1.020)]. PM_10_ showed a similar early-lag pattern, with significant associations from lag0 to lag2 and an RR of 1.009 (95% CI: 1.006, 1.013) at lag0. SO_2_ was significantly associated with CVD mortality from lag0 to lag2, with the largest estimate at lag1 [RR = 1.044 (95% CI: 1.018, 1.069)]. NO_2_ showed significant associations from lag0 to lag3 and at lag6, with an RR of 1.018 (95% CI: 1.011, 1.026) at lag0. O_3_-8h was also significantly associated with CVD mortality from lag0 to lag2, whereas CO showed significant associations mainly at lag0 and lag1. In the cumulative lag models (Figure 2B), PM_2.5_, PM_10_, SO_2_, NO_2_, and O_3_-8h showed significant cumulative associations across the examined lag windows. The cumulative estimate for PM_2.5_ peaked at lag0–2 [RR = 1.017 (95% CI: 1.010, 1.024)], and PM10 also reached its highest estimate at lag0–2 [RR = 1.011 (95% CI: 1.006, 1.016)]. SO_2_ showed the strongest cumulative association at lag0–2 [RR = 1.063 (95% CI: 1.032, 1.096)], while NO_2_ showed a progressively increasing cumulative association, reaching an RR of 1.029 (95% CI: 1.017, 1.041) at lag0–7. O_3_-8h showed modest but statistically significant cumulative associations, with estimates remaining significant through lag0–7 [RR = 1.005 (95% CI: 1.000, 1.010)]. For CO, significant cumulative associations were observed only in the shorter windows from lag0 to lag0–2, with the highest estimate at lag0–1 [RR = 1.008 (95% CI: 1.002, 1.014)], whereas associations were no longer statistically significant in longer cumulative windows. The robustness of the main lag results was further supported by sensitivity analyses. Additional adjustment for public holidays, Chinese New Year, and the COVID-19 pandemic period yielded estimates that were highly similar to those from the main model across cumulative lag windows (Supplementary Table S1; Figure S1). Distributed lag model analyses over lag0-7 yielded broadly consistent lag patterns, supporting the main findings within a unified lag framework (Supplementary Table S2). After false discovery rate correction for multiple lag tests, the associations for PM_2.5_, PM_10_, SO_2_, and NO_2_ remained generally robust across the main cumulative lag windows (Supplementary Table S1).

### Exposure-response curve analysis

**Figure 3:**
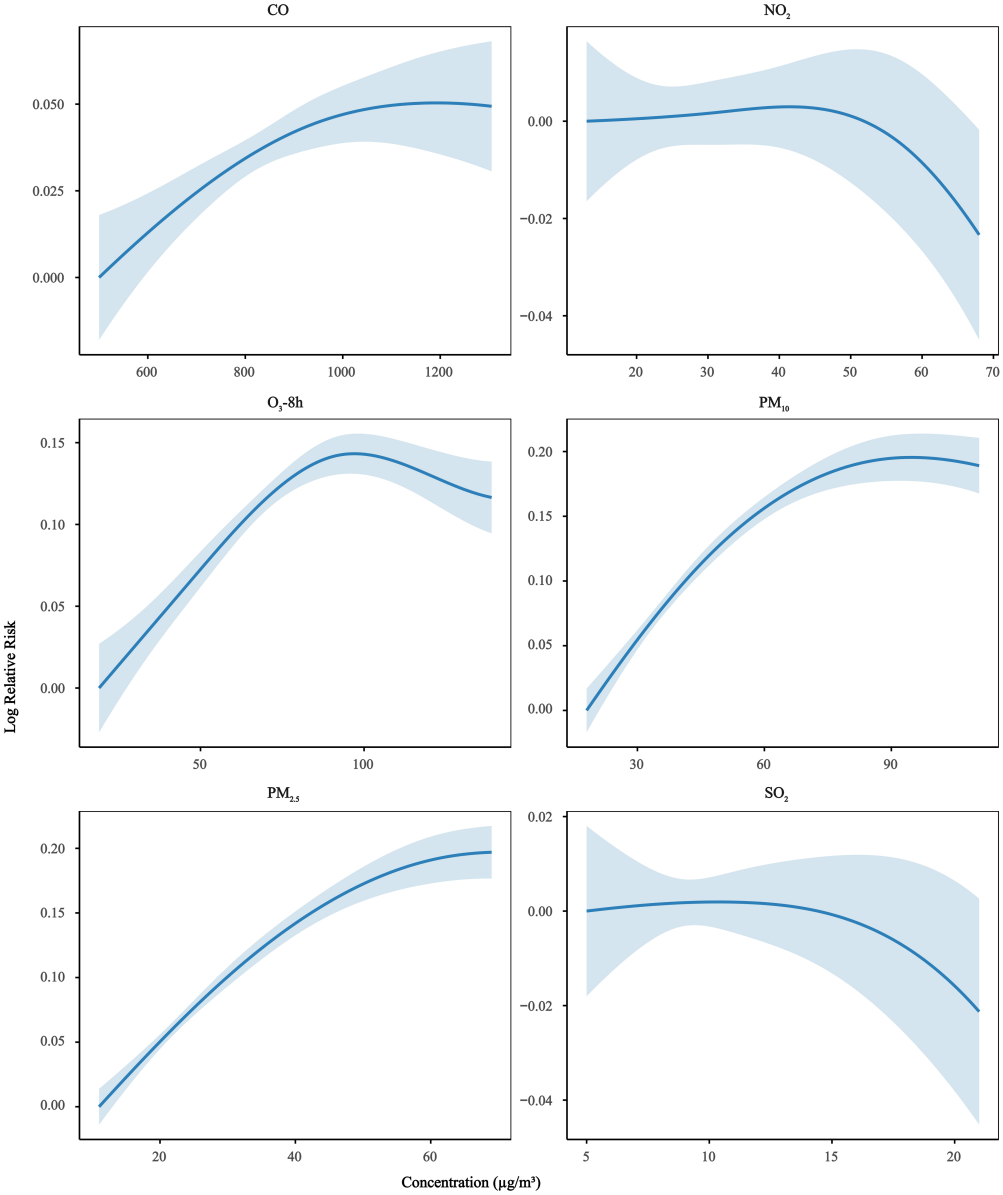
Exposure-response curves for the associations between individual air pollutants and cardiovascular mortality risk in single-pollutant models.

Figure 3 illustrates the exposure-response curves between air pollutant concentrations and the log-relative risk (log-RR) of CVD mortality. Non-linear relationships were observed for most pollutants across the studied concentration ranges. For particulate matter, the risk associated with PM_2.5_ and PM_10_ increased sharply at lower concentrations and exhibited a gradual attenuation in slope as concentrations exceeded approximately 50 μg/m^3^ and 80 μg/m^3^, respectively. A similar non-linear pattern was observed for O_3_-8h, where the risk rose progressively at lower levels, peaked at approximately 100 μg/m^3^, and subsequently showed a slight downward trend. Regarding gaseous pollutants, the risk for CO demonstrated a near-linear increase at concentrations below 1,000 μg/m^3^, followed by a plateau at higher levels. In contrast, the curves for NO_2_ and SO_2_ remained relatively stable with wide confidence intervals at lower concentrations. However, for both pollutants, a decrease in the log-RR was observed when concentrations exceeded approximately 45 μg/m^3^ for NO_2_ and 12 μg/m^3^ for SO_2._

### Effect modification by season and age

Stratified analyses by season revealed potential seasonal variation in pollutant effects. PM_2.5_ and PM_10_ were significantly associated with CVD mortality in both seasons. During the cold season, the ER% was 1.50% (95% CI: 0.85, 2.16%) for PM_2.5_ and 0.96% (95% CI: 0.51, 1.42%) for PM_10_; during the warm season, the corresponding estimates were 1.90% (95% CI: 0.58, 3.24%) and 1.07% (95% CI: 0.29, 1.87%), respectively. SO_2_ was significantly associated with CVD mortality only during the warm season [ER%: 5.61% (95% CI: 0.99, 10.44%)], whereas its cold-season estimate was not statistically significant [ER%: 2.50% (95% CI: −0.61, 5.71%)]. In contrast, NO_2_, O_3_-8h, and CO showed statistically significant associations only during the cold season, with ER% values of 1.94% (95% CI: 1.05, 2.84%), 0.64% (95% CI: 0.15, 1.13%), and 0.91% (95% CI: 0.28, 1.55%), respectively. However, none of the formal pollutant × season interaction tests reached statistical significance, with *p* values ranging from 0.223 to 0.800. Therefore, the season-stratified results suggest possible heterogeneity in point estimates, but do not provide strong statistical evidence for seasonal effect modification (Table 3).

**Table 3:** Season-stratified associations of ambient air pollutants with cardiovascular mortality and tests for seasonal effect modification.

Pollutant	Whole year	Cold season	Warm season	*p* for interaction
PM_2.5_	1.55 (0.95, 2.16)***	1.50 (0.85, 2.16)***	1.90 (0.58, 3.24)**	0.589
PM_10_	0.96 (0.55, 1.37)***	0.96 (0.51, 1.42)***	1.07 (0.29, 1.87)**	0.800
SO_2_	3.37 (0.61, 6.21)*	2.50 (−0.61, 5.71)	5.61 (0.99, 10.44)*	0.241
NO_2_	1.66 (0.89, 2.43)***	1.94 (1.05, 2.84)***	1.10 (−0.21, 2.43)	0.281
O_3_-8h	0.45 (0.06, 0.85)*	0.64 (0.15, 1.13)**	0.26 (−0.23, 0.76)	0.223
CO	0.84 (0.29, 1.38)**	0.91 (0.28, 1.55)**	0.66 (−0.34, 1.67)	0.672

Results are presented as ER% (95% CI). Cold season was defined as November to April and warm season as May to October. Models included pollutant × season interaction terms and were adjusted for long-term trend, daily mean temperature, relative humidity, and day of week. ER% was calculated per 10 μg/m^3^ increase in PM_2.5_, PM_10_, SO_2_, NO_2_, and O_3_-8h, and per 0.1 mg/m^3^ increase in CO. P for interaction refers to the *p*-value for the pollutant × season interaction term. *p < 0.05; **p < 0.01; ***p < 0.001.

Age-stratified analyses indicated differential susceptibility across age groups. Among individuals aged ≥65 years, all six pollutants were significantly associated with CVD mortality. Notably, the effect of NO_2_ was more pronounced in this group [ER%: 1.94% (95% CI: 1.25, 2.65%)] compared to those aged <65 years [ER%: 1.68% (95% CI: 0.43, 2.95%)], and O_3_-8h showed significance only in the older adults [ER%: 0.47% (95% CI: 0.13, 0.81%) vs. non-significant in the younger group. Conversely, among individuals aged <65 years, significant associations were observed only for PM_2.5_, PM_10_, and NO_2._ Interestingly, the effect estimate for PM_2.5_ was slightly higher in the younger group [ER%: 1.54% (95% CI: 0.57, 2.52%)] than in the older adults [ER%: 1.25% (95% CI: 0.71, 1.79%), though both were significant. For SO_2_, a significant association was found only in the ≥65 years group [ER%: 3.54% (95% CI: 1.07, 6.07%)], while the estimate for the <65 years group was imprecise and non-significant (Table 4).

**Table 4:** Excess risk (%) of cardiovascular mortality associated with air pollution by age group.

Pollutant	Total	Age <65	Age ≥65
CO	0.08 (0.04, 0.13)*	0.04 (−0.05, 0.13)	0.09 (0.04, 0.14)*
NO₂	1.88 (1.26, 2.50)*	1.68 (0.43, 2.95)*	1.94 (1.25, 2.65)*
O₃-8 h	0.43 (0.12, 0.74)*	0.49 (−0.02, 1.02)	0.47 (0.13, 0.81)*
SO₂	3.44 (1.24, 5.69)*	3.85 (−0.24, 8.13)	3.54 (1.07, 6.07)*
PM_10_	0.90 (0.58, 1.23)*	0.99 (0.35, 1.64)*	0.88 (0.51, 1.25)*
PM_2.5_	1.32 (0.83, 1.80)*	1.54 (0.57, 2.52)*	1.25 (0.71, 1.79)*

ER: excess risk per 10 μg/m^3^ increase in pollutant concentration, or per 0.1 mg/m^3^ increase for CO; values are presented as ER (%), 95% confidence interval (CI); All models adjusted for long-term trend, daily mean temperature, relative humidity, and day of week using natural cubic splines. *p < 0.05.

### Two-pollutant models and robustness checks

**Figure 4:**
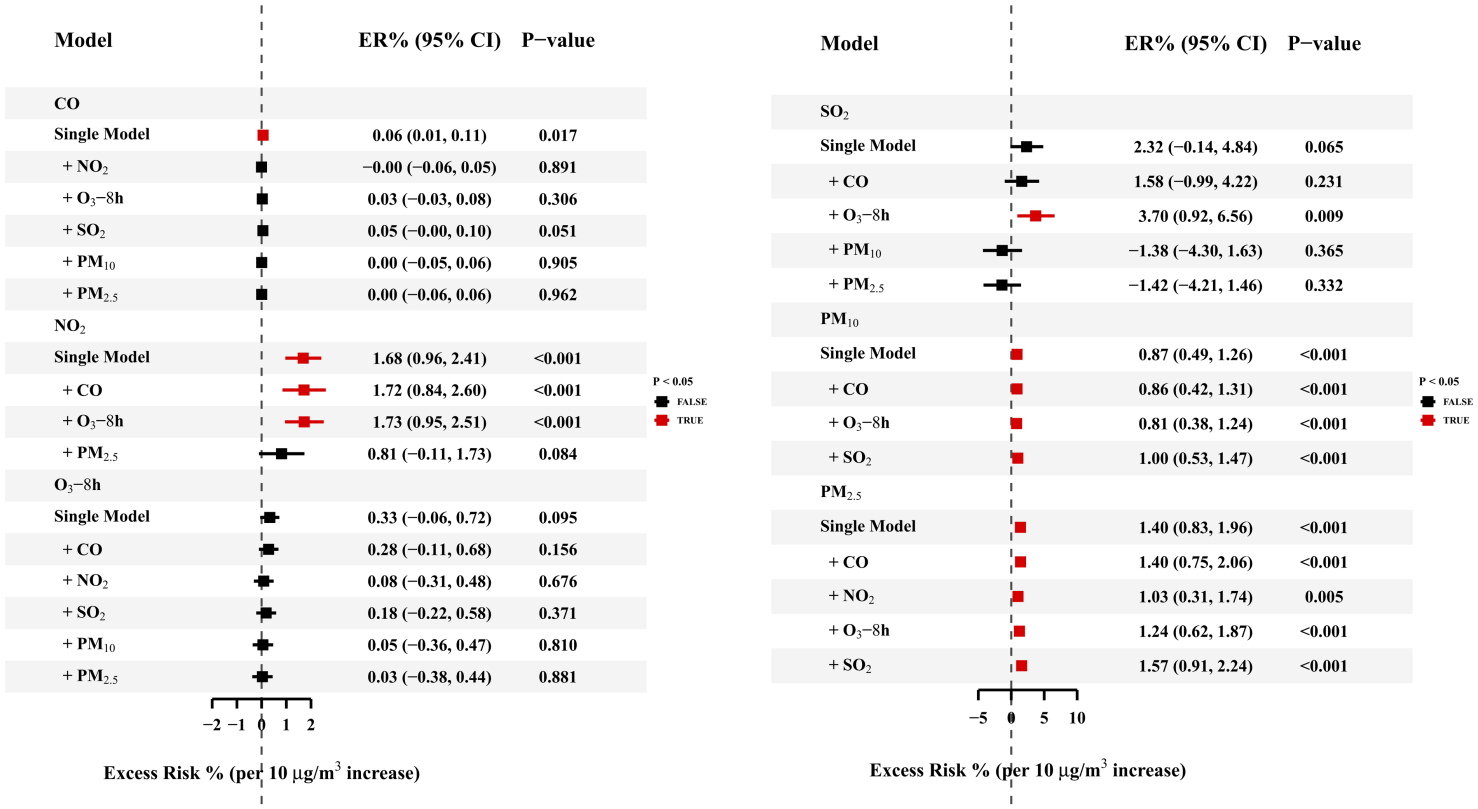
Excess risk percentages and 95% confidence intervals for cardiovascular mortality in two-pollutant models. Estimates are expressed per 10 μg/m^3^ increase in PM_2.5_, PM_10_, SO_2_, NO_2_, and O_3_-8h, and per 0.1 mg/m^3^ increase in CO.

The associations between air pollutants and CVD mortality in two-pollutant models are shown in Figure 4. The estimated risks for NO_2_, PM_2.5_, and PM_10_ persisted and remained statistically significant across all two-pollutant configurations. Specifically, the ER% for NO_2_ was 1.72% (95% CI: 0.84, 2.60%) when paired with CO, and 1.73% (95% CI: 0.95, 2.51%) when paired with O_3_-8h. For PM_2.5_, the ER% varied from 1.03% (95% CI: 0.31, 1.74%) in the model with NO_2_ to 1.57% (95% CI: 0.91, 2.24%) in the model with SO_2_. PM_10_ showed significant associations in all combinations, with ER% values ranging from 0.81% (95% CI: 0.38, 1.24%) to 1.00% (95% CI: 0.53, 1.47%). However, the association for SO_2_ became statistically non-significant when paired with PM_2.5_ (ER%: −1.42, 95% CI: −4.21, 1.46%) or PM_10_ (ER%: −1.38, 95% CI: −4.30, 1.63%). CO and O_3_-8h yielded no significant associations with CVD mortality in any of the two-pollutant models (*p* > 0.05). Collinearity diagnostics were further performed for the two-pollutant models. Consistent with the correlation matrix, PM_2.5_ and PM_10_ were highly correlated and were therefore not simultaneously included in the same two-pollutant model. For the included pollutant pairs, variance inflation factors and maximum condition indices indicated acceptable model stability (Supplementary Table S3).

**Figure 5:**
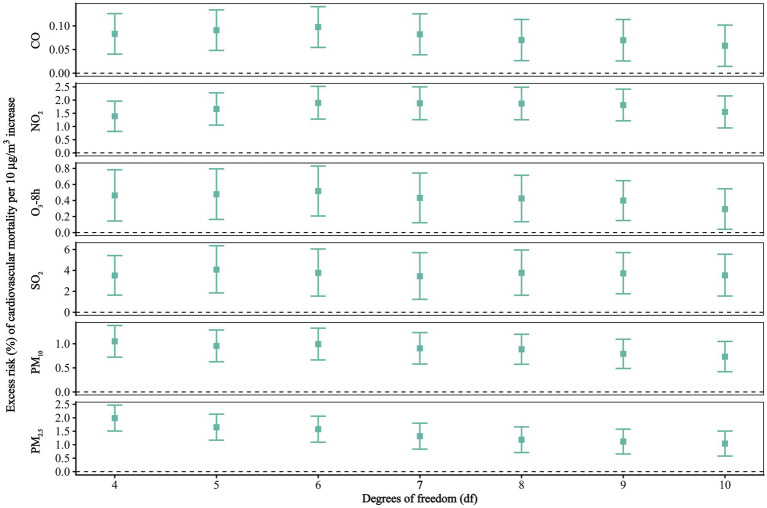
Sensitivity analysis of the effects of incremental concentrations of different air pollutants on cardiovascular disease mortality.

To assess the robustness of the model results, sensitivity analyses were conducted by varying the degrees of freedom (df) for the smoothing function of time trends. As the df for the time-series smoother varied from 4 to 10, the estimated ERs for all six pollutants remained largely invariant. The estimated effects of NO_2_, PM_2.5_, PM_10_, and O_3_-8h were highly stable across alternative degrees of freedom for the temporal trend. Notably, the effect estimates for SO_2_ remained the most pronounced throughout the range of df values (ER: 3.0–4.5%), showing minimal fluctuations and maintaining strong statistical significance in all scenarios. Although CO yielded the lowest absolute point estimates, its positive association with CVD mortality remained consistent regardless of the df specification (Figure 5). Extended lag analyses were conducted to evaluate potential mortality displacement. When the lag window was extended from lag0-7 to lag0-14 and lag0-21, the cumulative associations for PM_2.5_, PM_10_, SO_2_, NO_2_, and O_3_-8h showed no consistent attenuation. Distributed lag model analyses over lag0-21 also did not show a clear pattern of negative compensatory effects at later lags for these pollutants. In contrast, the estimates for CO became weaker and less stable over longer lag windows (Supplementary Table S4).

## Discussion

This 11-year (2014–2024) time-series study in Nanning, Guangxi, systematically evaluated the short-term associations between ambient air pollutants and daily CVD mortality. Overall, we found positive associations for short-term exposures to PM_2.5_, PM_10_, and NO_2_. Stratified analyses suggested broader susceptibility among older adults, whereas season-stratified estimates showed pollutant-specific variation without statistically significant pollutant-by-season interactions. Together, these findings add region-specific evidence on the CVD impacts of ambient air pollution in subtropical southern China.

The observed positive associations are broadly consistent with previous epidemiological evidence (1–3, 7, 20). In particular, the estimated short-term effect of PM_2.5_ on CVD mortality in this study falls within the range reported in major multi-city and nationwide analyses. For instance, a global study encompassing 652 cities documented risk increases ranging from 0.68 to 1.58% (11), while a large-scale analysis of 272 Chinese cities also confirmed significant short-term effects of PM_2.5_ (12). PM_10_ also showed a positive association, further supporting the substantial CVD burden associated with particulate pollution (5, 9, 10, 21, 22). From a biological perspective, inhaled fine particles may enter the circulation directly or trigger pulmonary inflammation (2). This process subsequently initiates a cascade of systemic oxidative stress and inflammation, leading to vascular endothelial dysfunction, destabilization of atherosclerotic plaques, and a pro-thrombotic state, ultimately precipitating acute cardiovascular events (7, 10, 23). In addition to inflammation-related mechanisms linked to particulate matter, NO_2_ has been associated with autonomic imbalance and reduced heart rate variability (24, 25), which may help explain its persistent association in the present study. In the two-pollutant models, the associations for PM_2.5_, PM_10_, and NO_2_ remained relatively stable across the eligible pollutant combinations, suggesting that these findings were less sensitive to co-pollutant adjustment. However, because PM_2.5_ and PM_10_ were highly correlated, their independent effects cannot be fully disentangled in the present analysis. Therefore, the particulate matter findings are better interpreted as reflecting the cardiovascular risk associated with the broader particulate pollution mixture rather than completely independent effects of each particulate fraction. Although SO_2_ showed a relatively large effect estimate in single-pollutant models, its association was attenuated after adjustment for particulate pollutants, suggesting that SO_2_ may partly reflect combustion-related pollution mixtures rather than a fully independent pollutant-specific effect.

Regarding O_3_-8h and CO, the overall evidence was less consistent than that for PM_2.5_, PM_10_, and NO_2_. O_3_-8h showed modest associations in early single-day and cumulative lag windows, whereas CO was mainly associated with CVD mortality in shorter lag windows and became less stable in longer cumulative and two-pollutant models. These patterns suggest that the cardiovascular effects of O_3_-8h and CO may be more sensitive to lag selection, population susceptibility, and co-pollutant adjustment. This finding is consistent with some studies where the O_3_ signal is sometimes attenuated when particulate matter is considered or exhibits more complex nonlinearity (4, 6), and where the short-term health association for CO may be less robust in areas with low background concentrations or due to its shorter atmospheric lifetime (26). However, our stratified analysis revealed a significant association for O_3_-8h specifically in the older adults, indicating their particular susceptibility, which is consistent with the biological hypothesis of diminished antioxidant defense and reduced pulmonary clearance in aging (1, 27).

The exposure-response curves provide additional insight into the observed associations. For PM_2.5_, PM_10_, and CO, the estimated risk increased approximately monotonically across much of the observed concentration range, without clear evidence of a threshold. This pattern supports the view that further reductions in current pollution levels may still yield public health benefits (28) and is also consistent with prior evidence suggesting that air pollution can remain harmful even at relatively low concentrations (4, 11). While the risk for CO demonstrated a near-linear increase at lower ranges, this should be interpreted with caution given the lack of overall statistical significance in the primary analysis. By contrast, the NO_2_ curve showed a decelerating increase at higher concentrations, whereas the SO_2_ curve suggested a steeper rise at lower concentrations. These findings may reflect differences in biological response, exposure misclassification, or residual confounding across concentration ranges (29). Extended lag analyses were further conducted to assess potential mortality displacement. The cumulative associations for PM_2.5_, PM_10_, SO_2_, NO_2_, and O_3_-8h showed no consistent attenuation over lag0–14 and lag0–21, and no clear negative compensatory pattern was observed at later lags. These findings suggest that the main pollutant associations were unlikely to be primarily explained by harvesting effects, although this possibility cannot be completely ruled out.

The season-stratified results suggested some variation in pollutant-specific estimates between the cold and warm seasons. However, the formal pollutant × season interaction tests were not statistically significant. Therefore, the observed seasonal differences should be interpreted as suggestive heterogeneity rather than definitive evidence of seasonal effect modification. Potential seasonal variation may be related to differences in pollutant mixtures, atmospheric stability, population activity patterns, and temperature-related cardiovascular stress (16, 30–32), although the present data do not support a statistically robust interaction between season and pollutant exposure.

Regarding population vulnerability, age stratification further delineated the at-risk groups. Individuals aged ≥65 years exhibited significantly higher susceptibility to all pollutants studied. This finding has a clear basis: aging is accompanied by decreased cardiovascular reserve, a high prevalence of underlying conditions (1, 15), and a decline in antioxidant defense and repair capacity (33, 34). Accordingly, several large cohort studies have confirmed the increased sensitivity of the older adults to air pollution. In contrast, the younger population (<65 years) was primarily susceptible to PM_2.5_, PM_10_, and NO_2_, while associations with SO_2_, O_3_-8h, and CO were not statistically significant. This differential susceptibility pattern strongly suggests that the older adults should be prioritized in protective policy development.

Several limitations should be acknowledged. First, exposure assessment was based on city-wide average pollutant concentrations from fixed-site monitoring stations rather than individual-level exposure measurements, which may have introduced non-differential exposure misclassification. Second, although we additionally adjusted for public holidays, Chinese New Year, and the COVID-19 pandemic period, data on influenza or other respiratory epidemics were not available; therefore, residual confounding by unmeasured time-varying factors cannot be fully excluded. Third, because the analysis used aggregated daily mortality counts, individual-level cardiovascular risk factors such as smoking, hypertension, diabetes, medication use, and socioeconomic status could not be adjusted for. Fourth, although the city-wide time-series design is appropriate for estimating acute population-level associations, it does not allow assessment of potential spatial heterogeneity within Nanning. Future studies using district-level mortality data, station-specific exposure estimates, or spatially varying models are warranted. Finally, although the extended lag analyses did not show clear evidence that the main associations were driven by mortality displacement, the possibility of harvesting effects cannot be completely ruled out.

## Conclusion

In conclusion, this 11-year time-series study demonstrates that short-term exposure to ambient air pollution is associated with increased CVD mortality in Nanning, with the most consistent evidence observed for PM_2.5_, PM_10_, and NO_2_. The associations across single-day and cumulative lag structures indicate that particulate and traffic-related air pollution may exert acute cardiovascular impacts in this subtropical urban setting. Stratified analyses further suggest that older adults represent a key vulnerable population during periods of elevated pollution. These findings provide locally relevant evidence for strengthening air quality management, health-risk communication, and early-warning strategies, and support targeted cardiovascular protection for vulnerable urban populations.

## Data Availability

The original contributions presented in the study are included in the article/Supplementary material, further inquiries can be directed to the corresponding authors.
