# Supplementary material for: Short-term associations between ambient air pollution and cardiovascular disease mortality: an 11-year time-series study in Nanning, China
Source: Front Public Health. 2026 Jul 15;14:1878398. doi: 10.3389/fpubh.2026.1878398 (PMC13416353; doi:10.3389/fpubh.2026.1878398)
Supplement: Supplementary file 1 [file Supplementary_file_1.docx]

| Table S1. Sensitivity analysis after additional adjustment for public holidays, Chinese New Year, and the COVID-19 pandemic period | | | | | | |
| --- | --- | --- | --- | --- | --- | --- |
| Pollutant | Cumulative lag | Main model ER% (95% CI) | Fully adjusted ER% (95% CI) | Change in ln(RR) (%) | Main FDR q | Fully adjusted FDR q |
| PM_2.5_ | lag0 | 1.37 (0.78, 1.96) | 1.38 (0.80, 1.97) | 1 | <0.001 | <0.001 |
|  | lag0-1 | 1.65 (0.99, 2.31) | 1.65 (1.00, 2.31) | 0.5 | <0.001 | <0.001 |
|  | lag0-2 | 1.68 (0.96, 2.40) | 1.68 (0.96, 2.40) | 0.1 | <0.001 | <0.001 |
|  | lag0-3 | 1.50 (0.72, 2.28) | 1.50 (0.72, 2.28) | -0.3 | <0.001 | <0.001 |
|  | lag0-4 | 1.19 (0.36, 2.02) | 1.19 (0.36, 2.03) | 0.4 | 0.008 | 0.009 |
|  | lag0-5 | 1.07 (0.19, 1.96) | 1.09 (0.21, 1.98) | 2.3 | 0.022 | 0.020 |
|  | lag0-6 | 1.17 (0.23, 2.11) | 1.22 (0.28, 2.16) | 4.3 | 0.018 | 0.015 |
|  | lag0-7 | 1.17 (0.18, 2.16) | 1.21 (0.23, 2.21) | 4 | 0.025 | 0.020 |
| PM_10_ | lag0 | 0.95 (0.56, 1.34) | 0.95 (0.58, 1.34) | 0.6 | <0.001 | <0.001 |
|  | lag0-1 | 1.08 (0.65, 1.52) | 1.07 (0.65, 1.50) | -0.9 | <0.001 | <0.001 |
|  | lag0-2 | 1.12 (0.65, 1.59) | 1.10 (0.63, 1.57) | -1.8 | <0.001 | <0.001 |
|  | lag0-3 | 1.03 (0.53, 1.54) | 1.01 (0.51, 1.51) | -2.5 | <0.001 | <0.001 |
|  | lag0-4 | 0.84 (0.30, 1.37) | 0.81 (0.28, 1.35) | -2.8 | 0.004 | 0.005 |
|  | lag0-5 | 0.76 (0.19, 1.32) | 0.74 (0.18, 1.30) | -1.9 | 0.012 | 0.015 |
|  | lag0-6 | 0.81 (0.22, 1.40) | 0.81 (0.22, 1.40) | -0.2 | 0.010 | 0.013 |
|  | lag0-7 | 0.83 (0.21, 1.45) | 0.83 (0.21, 1.45) | 0.1 | 0.012 | 0.015 |
| SO_2_ | lag0 | 3.95 (1.29, 6.68) | 3.50 (0.86, 6.20) | -11.2 | 0.007 | 0.015 |
|  | lag0-1 | 5.54 (2.60, 8.57) | 5.02 (2.11, 8.02) | -9.1 | <0.001 | 0.002 |
|  | lag0-2 | 6.35 (3.16, 9.63) | 5.73 (2.57, 8.99) | -9.4 | <0.001 | <0.001 |
|  | lag0-3 | 5.84 (2.45, 9.36) | 5.13 (1.76, 8.62) | -11.9 | 0.001 | 0.005 |
|  | lag0-4 | 5.22 (1.64, 8.92) | 4.44 (0.89, 8.12) | -14.6 | 0.007 | 0.019 |
|  | lag0-5 | 5.10 (1.36, 8.99) | 4.30 (0.58, 8.15) | -15.5 | 0.010 | 0.027 |
|  | lag0-6 | 5.66 (1.73, 9.74) | 4.84 (0.94, 8.89) | -14.2 | 0.007 | 0.019 |
|  | lag0-7 | 6.25 (2.12, 10.54) | 5.35 (1.26, 9.61) | -13.9 | 0.005 | 0.015 |
| NO_2_ | lag0 | 1.81 (1.05, 2.57) | 1.68 (0.91, 2.45) | -7.2 | <0.001 | <0.001 |
|  | lag0-1 | 2.19 (1.38, 3.02) | 2.05 (1.21, 2.89) | -6.5 | <0.001 | <0.001 |
|  | lag0-2 | 2.50 (1.62, 3.38) | 2.33 (1.42, 3.24) | -6.8 | <0.001 | <0.001 |
|  | lag0-3 | 2.64 (1.71, 3.59) | 2.43 (1.47, 3.41) | -7.9 | <0.001 | <0.001 |
|  | lag0-4 | 2.58 (1.59, 3.58) | 2.32 (1.30, 3.35) | -9.8 | <0.001 | <0.001 |
|  | lag0-5 | 2.60 (1.55, 3.65) | 2.31 (1.23, 3.40) | -10.8 | <0.001 | <0.001 |
|  | lag0-6 | 2.77 (1.66, 3.89) | 2.48 (1.34, 3.63) | -10.3 | <0.001 | <0.001 |
|  | lag0-7 | 2.91 (1.74, 4.10) | 2.63 (1.43, 3.84) | -9.8 | <0.001 | <0.001 |
| O_3_-8h | lag0 | 0.51 (0.16, 0.87) | 0.55 (0.23, 0.87) | 7.2 | 0.007 | 0.002 |
|  | lag0-1 | 0.76 (0.40, 1.11) | 0.76 (0.43, 1.10) | 0.7 | <0.001 | <0.001 |
|  | lag0-2 | 0.76 (0.36, 1.15) | 0.78 (0.40, 1.16) | 3 | <0.001 | <0.001 |
|  | lag0-3 | 0.72 (0.31, 1.13) | 0.74 (0.34, 1.15) | 3.2 | 0.001 | <0.001 |
|  | lag0-4 | 0.63 (0.20, 1.06) | 0.66 (0.23, 1.08) | 4.1 | 0.007 | 0.005 |
|  | lag0-5 | 0.50 (0.06, 0.95) | 0.53 (0.09, 0.98) | 6.1 | 0.031 | 0.021 |
|  | lag0-6 | 0.47 (0.01, 0.93) | 0.51 (0.06, 0.97) | 8.5 | 0.049 | 0.032 |
|  | lag0-7 | 0.50 (0.03, 0.97) | 0.55 (0.08, 1.02) | 10.2 | 0.043 | 0.026 |
| CO | lag0 | 0.75 (0.22, 1.29) | 0.70 (0.17, 1.24) | -6.2 | 0.009 | 0.015 |
|  | lag0-1 | 0.80 (0.22, 1.39) | 0.75 (0.17, 1.33) | -7.1 | 0.010 | 0.016 |
|  | lag0-2 | 0.70 (0.08, 1.33) | 0.63 (0.01, 1.26) | -10.3 | 0.031 | 0.051 |
|  | lag0-3 | 0.55 (-0.11, 1.21) | 0.47 (-0.19, 1.13) | -15 | 0.114 | 0.183 |
|  | lag0-4 | 0.30 (-0.40, 1.01) | 0.22 (-0.48, 0.92) | -27.3 | 0.428 | 0.577 |
|  | lag0-5 | 0.15 (-0.59, 0.90) | 0.08 (-0.66, 0.82) | -47 | 0.685 | 0.830 |
|  | lag0-6 | 0.21 (-0.57, 1.00) | 0.16 (-0.62, 0.94) | -26.3 | 0.603 | 0.706 |
|  | lag0-7 | 0.23 (-0.59, 1.06) | 0.19 (-0.63, 1.01) | -18.3 | 0.603 | 0.685 |
| Note: The main model, adjusted for long-term trend, temperature, relative humidity, and day of week, with the fully adjusted model, which additionally included public holidays, Chinese New Year, and the COVID-19 pandemic period; estimates are presented as ER% (95% CI) per 10 μg/m³ increase in PM_2.5_, PM_10_, SO_2_, NO_2,_ and O_3_-8h and per 0.1 mg/m³ increase in CO, and the percentage change in ln(RR) was calculated as [ln(RR_fully adjusted) − ln(RR_main)] / \|ln(RR_main)\| × 100, with negative values indicating attenuation after additional adjustment. | | | | | | |

| Table S2. Distributed lag model sensitivity analysis | | | | | | |
| --- | --- | --- | --- | --- | --- | --- |
| Pollutant | Lag | RR (95% CI) | ER% (95% CI) | Cumulative lag | RR (95% CI) | ER% (95% CI) |
| PM_2.5_ | lag0 | 1.011 (1.007-1.015) | 1.14 (0.72, 1.55) | lag0 | 1.011 (1.007-1.015) | 1.14 (0.72, 1.55) |
|  | lag1 | 1.006 (1.004-1.009) | 0.64 (0.41, 0.87) | lag0-1 | 1.018 (1.012-1.024) | 1.79 (1.15, 2.42) |
|  | lag2 | 1.002 (1-1.004) | 0.21 (0.04, 0.39) | lag0-2 | 1.02 (1.013-1.027) | 2 (1.28, 2.73) |
|  | lag3 | 0.999 (0.997-1.001) | -0.08 (-0.3, 0.15) | lag0-3 | 1.019 (1.012-1.027) | 1.92 (1.17, 2.69) |
|  | lag4 | 0.998 (0.996-1) | -0.18 (-0.4, 0.05) | lag0-4 | 1.017 (1.009-1.026) | 1.74 (0.93, 2.57) |
|  | lag5 | 0.999 (0.997-1.001) | -0.08 (-0.25, 0.09) | lag0-5 | 1.017 (1.008-1.025) | 1.66 (0.79, 2.55) |
|  | lag6 | 1.001 (0.999-1.004) | 0.15 (-0.06, 0.36) | lag0-6 | 1.018 (1.009-1.027) | 1.81 (0.89, 2.74) |
|  | lag7 | 1.004 (1.001-1.008) | 0.44 (0.06, 0.83) | lag0-7 | 1.023 (1.012-1.033) | 2.26 (1.21, 3.32) |
| PM_10_ | lag0 | 1.007 (1.004-1.01) | 0.71 (0.43, 0.98) | lag0 | 1.007 (1.004-1.01) | 0.71 (0.43, 0.98) |
|  | lag1 | 1.004 (1.002-1.006) | 0.4 (0.25, 0.56) | lag0-1 | 1.011 (1.007-1.015) | 1.11 (0.69, 1.54) |
|  | lag2 | 1.001 (1-1.003) | 0.14 (0.02, 0.25) | lag0-2 | 1.013 (1.008-1.017) | 1.25 (0.77, 1.73) |
|  | lag3 | 1 (0.998-1.001) | -0.04 (-0.19, 0.1) | lag0-3 | 1.012 (1.007-1.017) | 1.21 (0.71, 1.71) |
|  | lag4 | 0.999 (0.997-1) | -0.11 (-0.25, 0.04) | lag0-4 | 1.011 (1.006-1.016) | 1.1 (0.57, 1.63) |
|  | lag5 | 1 (0.998-1.001) | -0.05 (-0.16, 0.06) | lag0-5 | 1.011 (1.005-1.016) | 1.05 (0.49, 1.62) |
|  | lag6 | 1.001 (1-1.002) | 0.09 (-0.04, 0.23) | lag0-6 | 1.011 (1.006-1.017) | 1.14 (0.56, 1.74) |
|  | lag7 | 1.003 (1-1.005) | 0.27 (0.02, 0.52) | lag0-7 | 1.014 (1.008-1.021) | 1.42 (0.76, 2.09) |
| SO_2_ | lag0 | 1.032 (1.013-1.052) | 3.23 (1.28, 5.21) | lag0 | 1.032 (1.013-1.052) | 3.23 (1.28, 5.21) |
|  | lag1 | 1.018 (1.007-1.029) | 1.79 (0.74, 2.85) | lag0-1 | 1.051 (1.021-1.081) | 5.08 (2.1, 8.14) |
|  | lag2 | 1.006 (0.998-1.014) | 0.59 (-0.19, 1.37) | lag0-2 | 1.057 (1.024-1.091) | 5.69 (2.38, 9.12) |
|  | lag3 | 0.998 (0.988-1.008) | -0.19 (-1.21, 0.84) | lag0-3 | 1.055 (1.021-1.09) | 5.49 (2.1, 9) |
|  | lag4 | 0.997 (0.986-1.007) | -0.35 (-1.38, 0.69) | lag0-4 | 1.051 (1.016-1.088) | 5.12 (1.56, 8.81) |
|  | lag5 | 1.001 (0.993-1.009) | 0.11 (-0.66, 0.88) | lag0-5 | 1.052 (1.015-1.091) | 5.24 (1.47, 9.15) |
|  | lag6 | 1.01 (1.001-1.019) | 0.99 (0.07, 1.92) | lag0-6 | 1.063 (1.024-1.103) | 6.28 (2.36, 10.34) |
|  | lag7 | 1.021 (1.004-1.039) | 2.09 (0.35, 3.86) | lag0-7 | 1.085 (1.041-1.131) | 8.5 (4.05, 13.14) |
| NO_2_ | lag0 | 1.011 (1.006-1.017) | 1.12 (0.58, 1.67) | lag0 | 1.011 (1.006-1.017) | 1.12 (0.58, 1.67) |
|  | lag1 | 1.007 (1.004-1.01) | 0.73 (0.44, 1.02) | lag0-1 | 1.019 (1.01-1.027) | 1.86 (1.05, 2.68) |
|  | lag2 | 1.004 (1.002-1.006) | 0.39 (0.17, 0.61) | lag0-2 | 1.023 (1.014-1.032) | 2.25 (1.36, 3.16) |
|  | lag3 | 1.001 (0.998-1.004) | 0.15 (-0.15, 0.44) | lag0-3 | 1.024 (1.015-1.033) | 2.4 (1.49, 3.32) |
|  | lag4 | 1.001 (0.998-1.004) | 0.06 (-0.24, 0.36) | lag0-4 | 1.025 (1.015-1.034) | 2.46 (1.49, 3.44) |
|  | lag5 | 1.001 (0.999-1.003) | 0.12 (-0.1, 0.34) | lag0-5 | 1.026 (1.015-1.036) | 2.58 (1.54, 3.63) |
|  | lag6 | 1.003 (1-1.005) | 0.28 (0.01, 0.55) | lag0-6 | 1.029 (1.018-1.04) | 2.87 (1.79, 3.96) |
|  | lag7 | 1.005 (1-1.01) | 0.49 (-0.01, 1) | lag0-7 | 1.034 (1.021-1.046) | 3.37 (2.14, 4.62) |
| O_3_-8h | lag0 | 1.004 (1.002-1.007) | 0.42 (0.17, 0.67) | lag0 | 1.004 (1.002-1.007) | 0.42 (0.17, 0.67) |
|  | lag1 | 1.003 (1.001-1.004) | 0.26 (0.12, 0.4) | lag0-1 | 1.007 (1.003-1.011) | 0.68 (0.3, 1.07) |
|  | lag2 | 1.001 (1-1.002) | 0.12 (0.02, 0.21) | lag0-2 | 1.008 (1.004-1.012) | 0.8 (0.37, 1.23) |
|  | lag3 | 1 (0.999-1.001) | 0.01 (-0.11, 0.13) | lag0-3 | 1.008 (1.004-1.012) | 0.81 (0.37, 1.25) |
|  | lag4 | 1 (0.998-1.001) | -0.04 (-0.16, 0.08) | lag0-4 | 1.008 (1.003-1.012) | 0.77 (0.32, 1.22) |
|  | lag5 | 1 (0.999-1.001) | -0.03 (-0.12, 0.06) | lag0-5 | 1.007 (1.003-1.012) | 0.74 (0.28, 1.2) |
|  | lag6 | 1 (0.999-1.001) | 0.02 (-0.09, 0.12) | lag0-6 | 1.008 (1.003-1.012) | 0.76 (0.29, 1.22) |
|  | lag7 | 1.001 (0.999-1.003) | 0.08 (-0.12, 0.28) | lag0-7 | 1.008 (1.003-1.014) | 0.84 (0.32, 1.35) |
| CO | lag0 | 1.007 (1.003-1.011) | 0.7 (0.32, 1.08) | lag0 | 1.007 (1.003-1.011) | 0.7 (0.32, 1.08) |
|  | lag1 | 1.003 (1.001-1.005) | 0.31 (0.11, 0.52) | lag0-1 | 1.01 (1.004-1.016) | 1.01 (0.44, 1.59) |
|  | lag2 | 1 (0.998-1.001) | -0.01 (-0.17, 0.14) | lag0-2 | 1.01 (1.004-1.016) | 1 (0.37, 1.63) |
|  | lag3 | 0.998 (0.996-1) | -0.23 (-0.44, -0.02) | lag0-3 | 1.008 (1.001-1.014) | 0.77 (0.12, 1.42) |
|  | lag4 | 0.997 (0.995-0.999) | -0.29 (-0.5, -0.08) | lag0-4 | 1.005 (0.998-1.012) | 0.48 (-0.21, 1.17) |
|  | lag5 | 0.998 (0.997-1) | -0.18 (-0.34, -0.03) | lag0-5 | 1.003 (0.996-1.01) | 0.29 (-0.44, 1.03) |
|  | lag6 | 1 (0.998-1.002) | 0.03 (-0.17, 0.22) | lag0-6 | 1.003 (0.996-1.011) | 0.32 (-0.44, 1.09) |
|  | lag7 | 1.003 (0.999-1.007) | 0.3 (-0.07, 0.66) | lag0-7 | 1.006 (0.997-1.015) | 0.62 (-0.25, 1.49) |
| Note: Estimates were obtained from distributed lag models with a linear exposure-response function and a natural cubic spline for the lag dimension over lag 0–7. Models were adjusted for long-term trend, temperature, relative humidity, and day of week. ER% (95% CI) is presented per 10 μg/m³ increase in PM_2.5_, PM_10_, SO_2_, NO_2_, and O_3_-8h, and per 0.1 mg/m³ increase in CO. | | | | | | |

| Table S3. Collinearity diagnostics for two-pollutant models | | | | | |
| --- | --- | --- | --- | --- | --- |
| Main pollutant | Co-pollutant | Spearman r | VIF for main pollutant | VIF for co-pollutant | Maximum condition index |
| PM_2.5_ | PM_10_ | 0.94 | 13.53 | 14.27 | 26.62 |
| PM_2.5_ | SO_2_ | 0.56 | 2.73 | 3.60 | 26.47 |
| PM_2.5_ | NO_2_ | 0.67 | 3.30 | 4.04 | 26.67 |
| PM_2.5_ | O_3_-8h | 0.38 | 2.20 | 3.42 | 25.71 |
| PM_2.5_ | CO | 0.49 | 2.57 | 3.28 | 26.79 |
| PM_10_ | SO_2_ | 0.58 | 3.04 | 3.81 | 26.41 |
| PM_10_ | NO_2_ | 0.70 | 4.25 | 4.94 | 26.60 |
| PM_10_ | O_3_-8h | 0.44 | 2.34 | 3.46 | 25.68 |
| PM_10_ | CO | 0.42 | 2.78 | 3.36 | 26.69 |
| SO_2_ | NO_2_ | 0.58 | 3.64 | 3.37 | 26.45 |
| SO_2_ | O_3_-8h | 0.26 | 2.69 | 3.17 | 25.36 |
| SO_2_ | CO | 0.40 | 2.75 | 2.66 | 26.57 |
| NO_2_ | O_3_-8h | 0.12 | 2.59 | 3.29 | 25.66 |
| NO_2_ | CO | 0.53 | 3.38 | 3.52 | 26.76 |
| O_3_-8h | CO | -0.09 | 3.08 | 2.53 | 25.95 |

| Table S4. Extended-lag sensitivity analysis for potential mortality displacement | | | |
| --- | --- | --- | --- |
| Pollutant | lag0–7 ER% (95% CI) | lag0–14 ER% (95% CI) | lag0–21 ER% (95% CI) |
| PM_2.5_ | 2.16 (1.11, 3.21) | 2.45 (0.98, 3.93) | 2.98 (1.00, 4.99) |
| PM_10_ | 1.36 (0.71, 2.01) | 1.65 (0.78, 2.52) | 2.17 (1.00, 3.34) |
| SO_2_ | 8.38 (4.08, 12.86) | 12.95 (7.17, 19.05) | 19.41 (11.41, 27.98) |
| NO_2_ | 3.71 (2.52, 4.92) | 5.17 (3.50, 6.87) | 6.31 (4.05, 8.61) |
| O_3_-8h | 0.75 (0.25, 1.25) | 0.84 (0.23, 1.46) | 0.99 (0.17, 1.81) |
| CO | 0.29 (-0.55, 1.13) | -0.34 (-1.48, 0.82) | -0.83 (-2.38, 0.74) |
| Note: Models were adjusted for long-term trend, daily mean temperature, relative humidity, and day of week. ER% was calculated per 10 μg/m³ increase in PM_2.5_, PM_10_, SO_2_, NO_2_, and O_3_-8h, and per 0.1 mg/m³ increase in CO. | | | |


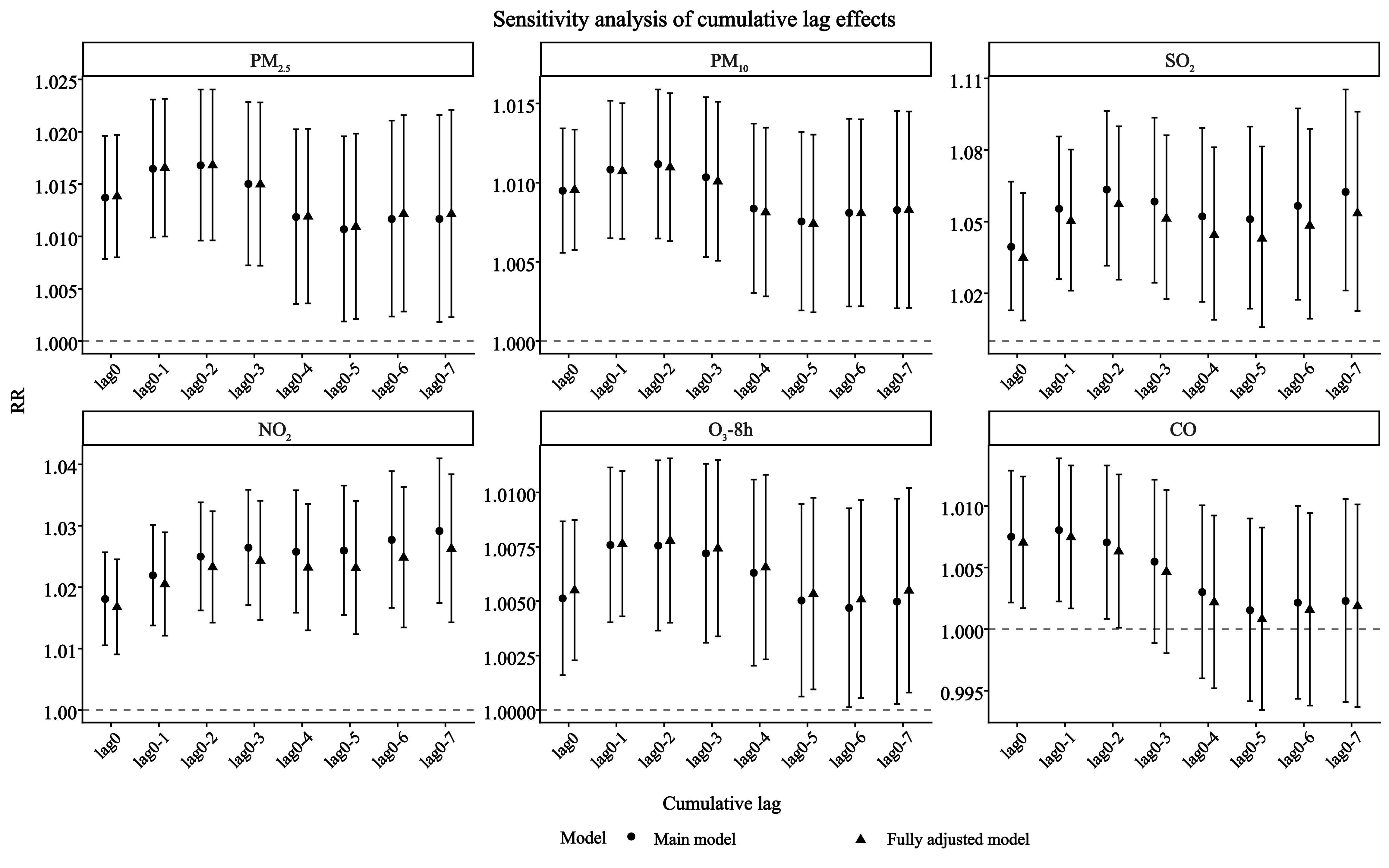


Figure S1. Sensitivity analysis of cumulative lag effects of ambient air pollutants on cardiovascular mortality. Relative risks (RRs) and 95% confidence intervals are shown for cumulative lag windows from lag0 to lag0-7. Circles represent the main model adjusted for long-term trend, temperature, relative humidity, and day of week; triangles represent the fully adjusted model additionally controlling for public holidays, Chinese New Year, and the COVID-19 pandemic period. Estimates are expressed per 10 μg/m³ increase in PM_2.5_, PM_10_, SO_2_, NO_2_, and O_3_-8h, and per 0.1 mg/m³ increase in CO.
